# Immersion infection of germ-free zebrafish with *Listeria monocytogenes* induces transient expression of innate immune response genes

**DOI:** 10.3389/fmicb.2015.00373

**Published:** 2015-04-29

**Authors:** Ying Shan, Chun Fang, Changyong Cheng, Yong Wang, Jinrong Peng, Weihuan Fang

**Affiliations:** ^1^Zhejiang Provincial Key Laboratory of Preventive Veterinary Medicine, Institute of Preventive Veterinary Medicine, Zhejiang UniversityHangzhou, China; ^2^Key Laboratory for Molecular Animal Nutrition, Ministry of Education, College of Animal Sciences, Zhejiang UniversityHangzhou, China; ^3^Molecular Microbiology and Food Safety Laboratory, Institute of Preventive Veterinary Medicine, College of Animal Sciences, Zhejiang UniversityHangzhou, China

**Keywords:** germ-free zebrafish, *Listeria monocytogenes*, infection model, immersion, innate immune responses

## Abstract

Zebrafish, *Denio rerio,* can be an alternative to other classic animal models for human infectious diseases to examine the processes of microbial infections and host–pathogen interactions *in vivo* because of their small body dimension but large clutch size. We established germ-free zebrafish infection models of *Listeria monocytogenes* through different routes of infection: oral immersion and injection via yolk sac, brain ventricle and blood island. Immersion of zebrafish larva even with 10^10^ CFU/mL *L. monocytogenes* EGDe strain in egg water was unable to cause mortality, but GFP-expressing bacteria in the gut lumen can be observed in frozen sections. Several selected maker genes of the innate immune system, including *cyp1a*, *irg1l*, *il1b,* and *mmp9*, were significantly induced by oral immersion not only with strain EGDe, but also with strain M7 and *L. innocua*, though to a lesser degree (*P* < 0.01). Such induction appears to be transient with peak at 48 h post-infection, but returned to basal level at 72 h post-infection. Of the three injection routes, mortality after infection by yolk sac was 80% in early stage of infection. Few eggs can survive and hatch. Injection into zebrafish embryos via brain ventricle or blood island led to progressive lethal infection. *L. mocytogenes* EGDe showed steady replication in the fish embryos and was far more pathogenic than strain M7, which is consistent with findings in the murine model. We conclude that zebrafish can serve as susceptible and microscopically visible infection models for *L. monocytogenes* via different routes and can be applied to further studies on the interactions between bacterial virulence factors and host immune responses.

## Introduction

Zebrafish, *Denio rerio*, as a hybrid animal model between invertebrate and vertebrate, has been widely used for studying human infectious diseases. Zebrafish embryos can be an alternative to other classic animal infection models to examine processes of microbial infections and host–pathogen interactions *in vivo* because of their small body dimension but large clutch size (Tobin et al., 2012). Zebrafish larva has an independent innate immune system. The adaptive immune system is immature until 4–6 weeks post fertilization (Trede et al., 2004). This developing stage during the first 4 week can be employed to study innate immunity to infections by pathogenic organisms.

Several infection techniques have been approached in zebrafish models for over 20 different pathogenic bacterial species (Stockhammer et al., 2009; Li and Hu, 2012) as well as several fungal and viral pathogens (Sanders et al., 2003; Chao et al., 2010). The most commonly used method is micro-injection via the blood island near the urogenital opening in the 26 hpf (hours post fertilization) zebrafish. With intravenous infection via blood island, zebrafish is susceptible to almost all bacterial pathogens used so far, including *Mycobacterium marinum*, *Salmonella typhmurium,* and *Escherichia coli* (Prouty et al., 2003; Davis and Ramakrishnan, 2009; Volkman et al., 2010). Other routes of infection include micro-injection into brain ventricle or yolk sac of fish embryos. The brain ventricle is a good site to observe macrophage migration upon intraventricular introduction of pathogens because it is a closed cavity lack of macrophages in the embryonic stage (Davis et al., 2002). Yolk sac injection is suitable for large-scale screening because of its easiness of operation (Carvalho et al., 2011).

Additional advantages of using zebrafish as infection models are that the fish can be readily made germ-free with simple methods (Pham et al., 2008) and that genetic manipulation is relatively easy to generate transgenic lines (Tobin et al., 2012) for some specific experiments to better understand the host–microbe interactions. Two methods were examined for colonizing the developing gut of 5 dpf (days post fertilization) germ-free zebrafish with a defined anaerobic microbial community derived from a single human fecal sample (Toh et al., 2013). Inoculation *per os* revealed a key role for adhesion in protection by probiotic bacteria in a zebrafish colonization model (Rendueles et al., 2012). These studies suggest that germ-free zebrafish can serve as a useful tool for studying the interaction between specific pathogen and host intestinal mucosa.

*Listeria monocytogenes*, when orally exposed, can break through the intestinal barrier and cause systemic infections, such as septicemia and meningitis. It has been used as a model intracellular organism in infection biology studies and its pathogenic mechanisms are well illuminated (Cossart, 2007; Stavru et al., 2011). *Listeria* strains can be isolated from fish (Rodas-Suárez et al., 2006). However, it remains unknown if fish can develop listeriosis by natural way. Adult zebrafish was found less susceptible than mice (Menudier et al., 1996). However, the experiments were conducted at 22°C, at which temperature *Listeria* virulence genes are expressed (Johansson et al., 2002). Zebrafish larva at 54 hpf was used for real-time analysis of responses of macrophages to listerial infection by intravenous route (Levraud et al., 2009). The group also indicated that intravenous infection was less lethal than yolk sac infection, and that immersion infection did not induce lethality, possibly indicating failure of the bacterium to cross the intestinal barrier. *Edwardsiella tarda*, a pathogen natural to aquatic species, presented variable lethality to zebrafish larva upon immersion infection (van Soest et al., 2011). The channel catfish pathogen *E. ictaluri* was highly pathogenic to zebrafish larvae as shown by high mortality within 3 days after immersion exposure (Rendueles et al., 2012). These studies suggest that pathogenic progression to systemic lethal infection varies with the pathogens used for immersion infection. However, these experiments were conducted on conventional zebrafish where there might be native microbial flora that can interfere with the outcome of infecting pathogens. Also few studies were conducted on germ-free zebrafish for virulence mechanisms of pathogenic bacteria by different routes of infection.

We attempted to use germ-free zebrafish embryos as infection models by multiple routes of inoculation to investigate the pathogenicity of *L. monocytogenes* strains and *L. innocua* that are known to be of different virulence in mammalian species. Expression of innate immune response genes was examined to study if immersion can induce immune responses in infected fish larvae. We found that immersion infection, though not lethal, can lead to induction of several genes related to innate immunity. Infections by multiple injection routes can clearly differentiate the pathogenic potentials of listerial strains as assessed by hatching rate, surviving larvae and bacterial burden in the body. Such models can be explored to examine the virulence factors of and host responses to pathogenic bacteria other than *Listeria*.

## Materials and Methods

### Bacterial Strains and Culture Condition

*Listeria monocytogenes* strain EGDe, strain M7 (a low pathogenic strain isolated from milk in our laboratory; Chen et al., 2010), and *L. innuoca* strain ATCC33090 were used for infection experiments. To observe the bacteria *in vivo*, GFP-expressing strains EGDe-gfp, M7-gfp and ATCC-gfp were generated by transforming the respective strains with the recombinant plasmid pFL251 carrying *gfp* under control of the listerial *dlt* promoter constructed in our laboratory using the shuttle vector pAM401 (a kind gift from Dr. Nancy E. Freitag) as the backbone. The *dlt* promoter (in front of dltA, D-alanyl-lipoteichoic acid, of the *dlt* operon; Fortineau et al., 2000) was PCR amplified from *L. monocytogenes* EGDe genome. Bacteria were grown in brain heart infusion (BHI, Oxoid, UK) medium at 37°C with shaking at 150 rpm.

### Zebrafish Husbandry and Generation of Germ-Free Embryos

The zebrafish line AB was provided by Professor JR Peng (Zhejiang University, China). Adult fish were raised in the standard zebrafish unit (Aisheng, Beijing, China) at 28°C under a constant light cycle of 14-h on/10-h off. Germ-free embryos were generated with the method previously described (Pham et al., 2008). Natural breeding eggs were collected immediately after hatching and transferred to a sterile dish with sterilized egg water containing antibiotics (ampicillin and kanamycin). Unfertilized embryos were removed timely over the next few days.

### Bacterial Immersion and Injection

Overnight bacterial cultures were washed with sterile egg water and adjusted to OD_600__nm_ at 0.6 (10^9^ CFU/mL). Infection was performed by oral immersion and by micro-injections via yolk sac, brain ventricle or blood island, as previously described (Jennifer and Hazel, 2009; Benard et al., 2012). For static immersion, naturally hatched germ-free fish 5 dpf (when mouth and gut are functional) were used. For yolk sac infection, an average of 11 CFU (range 8–14 CFU) of each listerial strain was micro-injected directly at one-cell stage immediately after fertilization. In models via injection (except for yolk sac infection), 26 hpf fish were dechorionated carefully with ophthalmic forceps and anesthetized. At this stage, they developed into the prim-stage forming closed cavity of brain ventricles and blood island. One nanoliter of each bacterial suspension (mixed with phenol red indicator and contained different concentration of live bacteria depending on the infection routes and experimental purpose) was injected. Effect of different levels of inoculum (EGDe from 11 to 1100 CFU per fish on average) on survival was examined on yet-to-hatch zebrafish by infection via blood island. Intraventricular or intravenous infection was also conducted with fixed inoculum, about 11 (8–14) or 110 (80–140) CFU. In all cases, injection with egg water was used as mock infection.

### RNA Isolation and Quantitative Reverse Transcription PCR

Ten embryos of each group were pooled and collected by centrifugation at 12,000 *g* for 10 min and stored at -80°C for later use. Embryos were homogenized in 500 μL lysis buffer and total RNA was extracted according to the Uniq-10 TRNzol total RNA Extraction and Purification Kit instruction (Tiangen, Beijing, China). DNaseI (Promega, USA) was used to remove residual genomic DNA at 37°C for 1 h before cDNA synthesis. Reverse transcriptase (TOYOBO, Japan) was used for cDNA synthesis. Quantitative reverse transcription PCR was then performed in a 20 μl reaction mixture containing SYBR quantitative PCR mix (TOYOBO, Japan) to measure transcriptional levels of immune related genes (*cyp1a*, *irg1l*, *il1b,* and *mmp9*) with specific primer pairs (**Table 1**) using an iCycler iQ5 real time PCR detection system (Bio-Rad, USA). The housekeeping gene β*-actin* was used as internal control for normalization of transcriptional levels of the target genes.

**Table 1:** Quantitative polymerase chain reaction primer sequences used in this study.

Primers	5′-3′	Sequence
mmp9	Forward	5′-CATTAAAGATGCCCTGATGTATCCC
	Reverse	5′-AGTGGTGGTCCGTGGTTGAG
il1b	Forward	5′-GAACAGAATGAAGCACATCAAACC
	Reverse	5′-ACGGCACTGAATCCACCAC
cyp1a	Forward	5′-CCATTCAGACATATCGTAGTATCC
	Reverse	5′-CGCACCAGTTCATCATCATC
Irg1	Forward	5′-GGTTAGAAGCAAGTCCTC
	Reverse	5′-TGTGTTCATCCTCCTCAG
β-actin	Forward	5′-CGAGCTGTCTTCCCATCCA
	Reverse	5′-TCACCAACGTAGCTGTCTTTCTG

### Bacterial Enumeration

Eight embryos in each group were anesthetized at different time points after intravenous injection. Each embryo was then rinsed and homogenized in 1 mL sterile egg water. For fish infected by immersion, trunks and intestines were separated by a sterile syringe needle (gage size 26) under the stereo microscope. Five trunks or intestines were pooled and homogenized in 100 μL sterile egg water. Serial dilutions in PBS (10 mM, pH 7.4) of the homogenates were plated on PALCAM agar (*Listeria* selective medium; Luqiao, Beijing, China). The colonies were enumerated after incubation at 37°C for 24 h. The results were presented as mean log_10_ CFU ± SE per five fish.

### Cryosection

Embryos were fixed in 4% paraformaldehyde for 1 h at room temperature, then washed three times in sterile egg water (each for 5 min) before being embedded in 1.5% agarose/30% sucrose, and mounted in a small chamber. The blocks were trimmed to the shape of a pyramid with a surgical blade and equilibrated in 30% sucrose for at least 1 day at 4°C. After equilibration, the pyramids were mounted in optimal cutting temperature (O.C.T.) compound (SAKURA) in plastic molds which were then brought onto dry ice for immediate freezing. Prior to cryosectioning, the blocks were mounted on the supporter with O.C.T. compound and equilibrated at -30°C in a pre-chilled microtome (Leica, HM505) for 2 h.

### Infection with EGFP-Expressing Listeria and Live Embryo Imaging

Zebrafish larvae were infected by injection with the EGDe-gfp strain via brain ventricles or blood island and observed microscopically at 6 hpi (brain infection) or at 6, 24, and 48 hpi (intravenous). The GFP-expressing bacteria were monitored and photographed *in vivo* by laser confocal microscope IX81-FV1000 (Olympus, Japan).

### Confirmation to the Relevant Regulatory Standards

All animal experiments in this study were approved by the Laboratory Animal Management Committee of Zhejiang University (Approval No. 2013038).

## Results

### Oral Infection Did Not Cause Death, but Induced Transcription of Innate Immunity Related Genes

**FIGURE 1:**
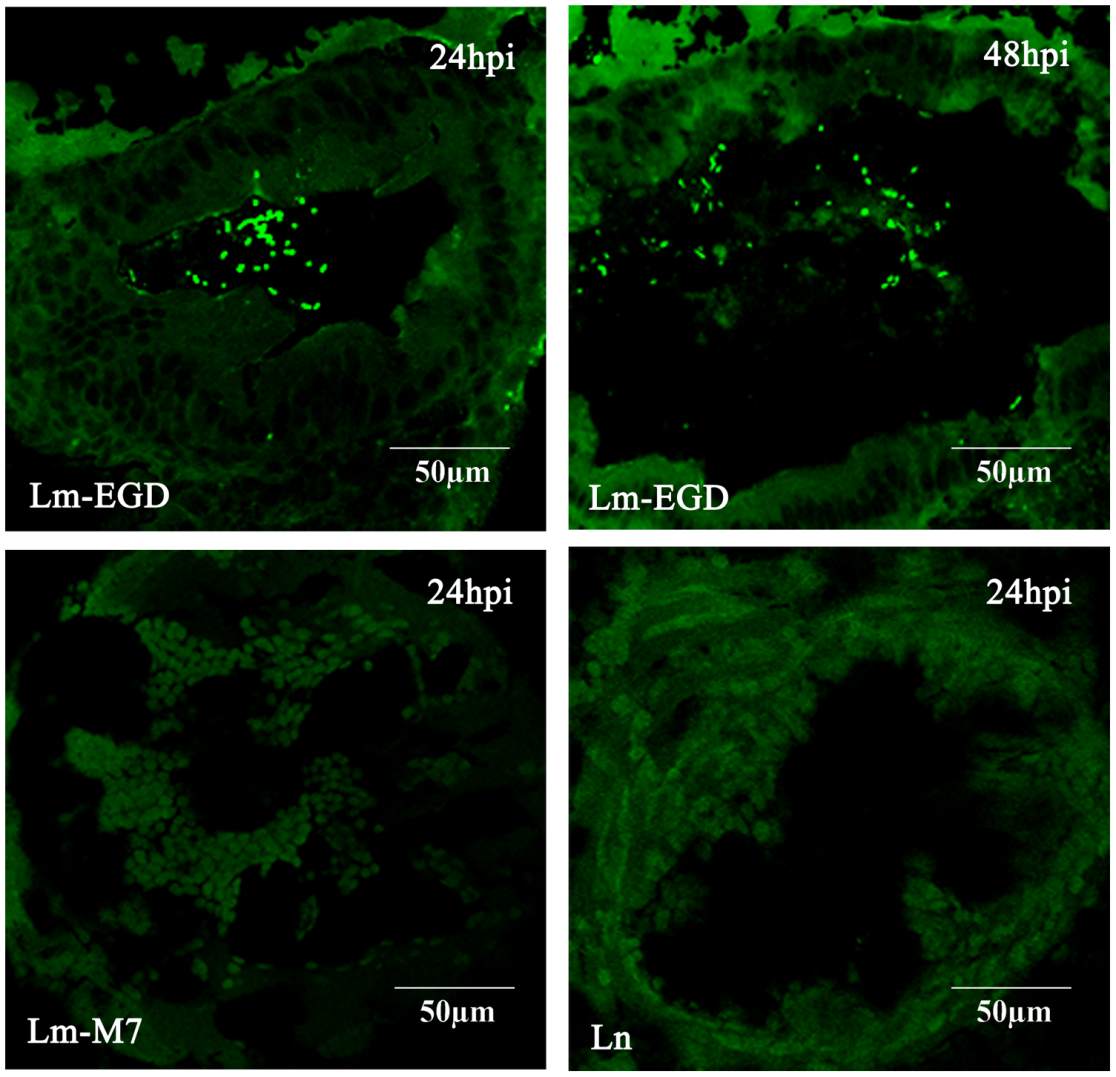
**Bacterial distribution in representative frozen sections of germ-free zebrafish infected at 5 dpf by 24 h of immersion with GFP-expressing listerial strain.** Images were taken with the confocal microscope in samples taken at 24 and 48 h post infection (hpi; five fish/strain at each time point). Lm-EGD and Lm-M7 stand for *L. monocytogenes* strains EGDe and M7 respectively; and Ln, for *L. innocua*. Same abbreviations are used for the following figures.

In order to mimic the natural route of infection through the digestive tract in mammalian species, we performed oral infection of germ-free fish by static immersion of different concentration of bacterial suspension of the EGDe strain. However, there was no death even with 10^10^ CFU/mL in egg water during the 10-days observation period. To test if the bacteria enter into the gut, we imaged sections of zebrafish 24, 48, and 72 dpi by confocal microscope and found that there were significant amount of EGDe-gfp bacteria in the gut lumen 24 and 48 hpi but not in any other tissues (**Figure 1**) while M7-gfp or ATCC-gfp can not be observed. In 72 hpi fish sections, bacteria was no longer observed in gut lumen.

**FIGURE 2:**
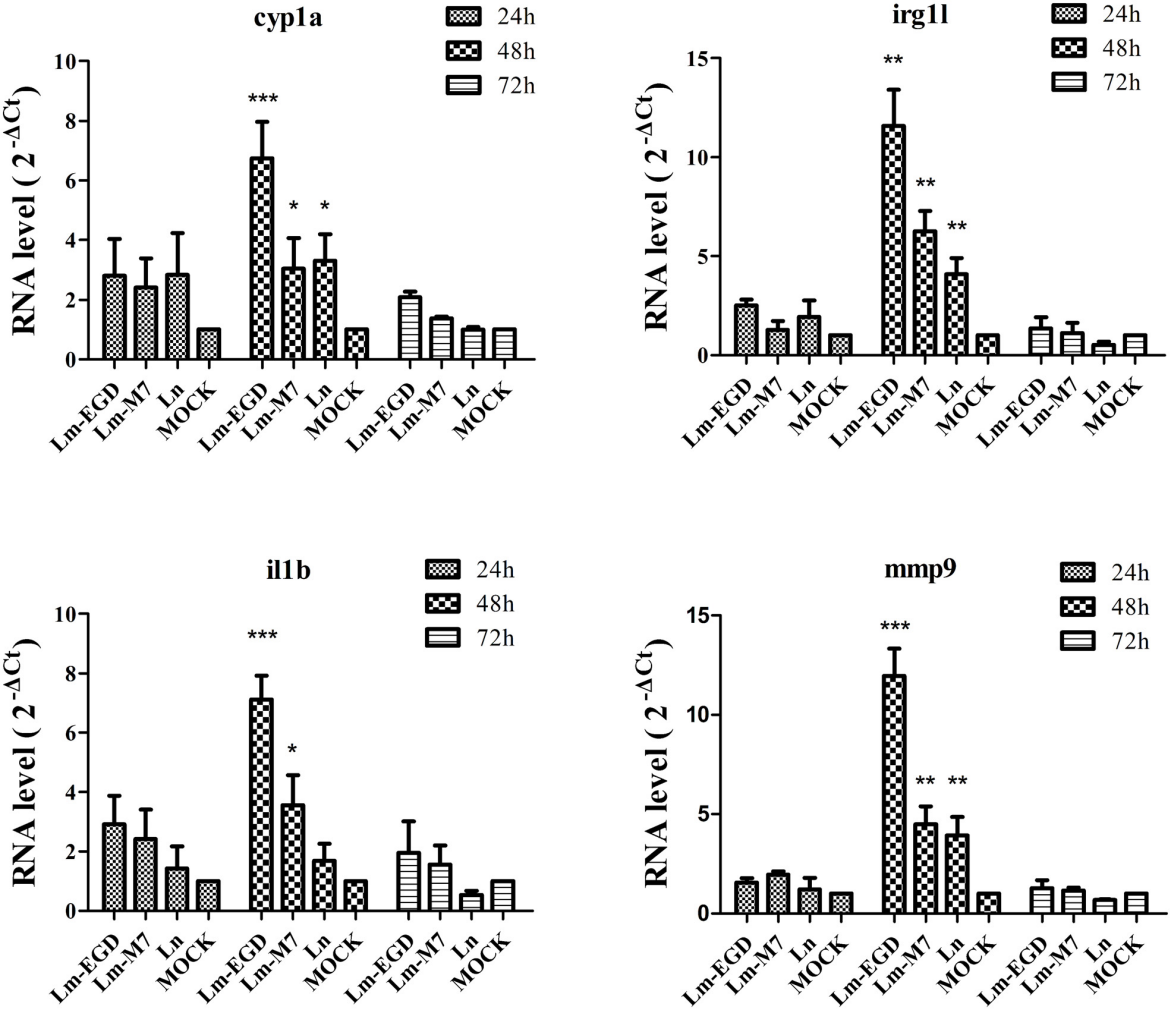
**Transcriptional analysis by qPCR of innate immune related genes *cyp1a*, *irg1l*, *il1b,* and *mmp9* of germ-free zebrafish infected at 5 dpf by 24 h of immersion with different listerial strains.** Data are expressed as mean ± SD of four independent experiments (each with 10 embryos pooled for total RNA extraction and qPCR). *P < 0.05, **P < 0.01 and ***P < 0.001.

**FIGURE 3:**
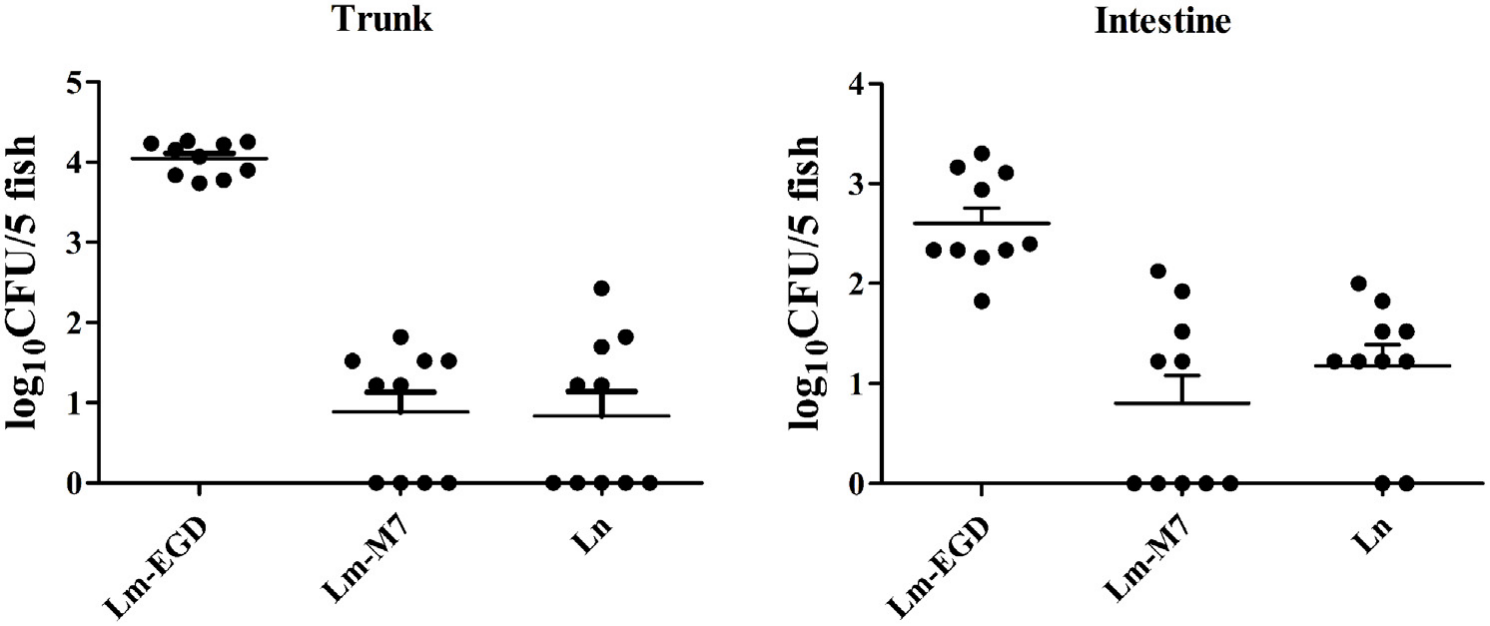
**Bacterial burden in the body trunk and intestine of *L. monocytogenes* EGDe infected germ-free fish was much higher than that of the strain M7 or *L. innocua* infected ones.** The fish (50 per strain) were immersion-infected at 5 dpf and sacrificed at 24 hpi. The body trunk and intestine samples of each strain were divided into 10 subgroups, each having five fish larvae to be homogenized and resuspended in 100 μl of PBS, from which a volume of 10-μl suspension was spotted in triplicate on the PALCAM agar plates for bacterial enumeration. Data were expressed as log_10_ CFU per five fish.

To examine immune responses to such non-lethal infection, the transcriptional levels of genes related to innate immunity *Cyp1a, Irg1l, Il1b,* and *Mmp9* were analyzed from fish at 24, 48, and 72 hpi. We found that these genes were significantly induced at 48 hpi but returned to the normal level at 72 hpi (**Figure 2**). Highest induction was observed in strain EGDe infected fish. These results were consistent with the findings by plate counting and confocal imaging that bacterial burden in EGDe exposed fish was much higher than that in M7 or *L. innocua* exposed fish both in the trunk and in intestine (**Figure 3**).

### Hatchability After Yolk Sac Injection was Reduced and the Reduction Varied with Strains

**FIGURE 4:**
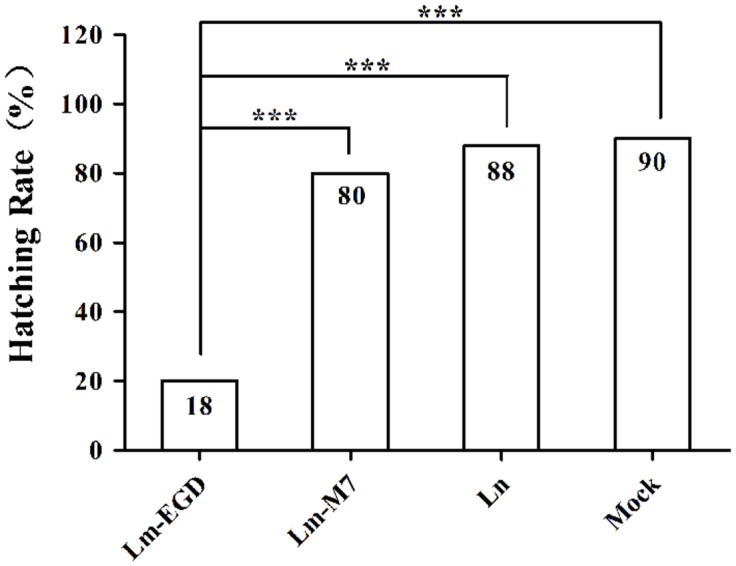
**Hatching rate of zebrafish eggs infected with different listerial strains (10 CFU per fish, *n* = 50) via yolk sac injection.** ***P < 0.001.

Infection with EGDe caused higher mortality (82%) with few eggs survived and hatched than the strain M7 (20%, *P* < 0.005; **Figure 4**) previously shown as low pathogenic in the mouse model (Chen et al., 2010). There was virtually no difference of hatchability between the *L. innuoca* and mock groups (88 vs. 90%), indicating the non-pathogenic nature of this listerial species.

### Survival Rate Differed Among Strains After Brain Ventricle Injection

**FIGURE 5:**
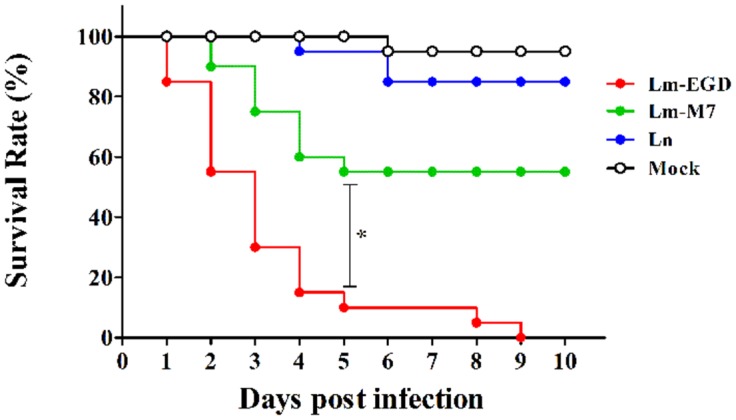
**Percent survival of 26 hpf germ-free zebrafish embryos (*n* = 20) infected with different listerial strains (100 CFU per fish) via brain ventricle injection.** *P < 0.05.

**FIGURE 6:**
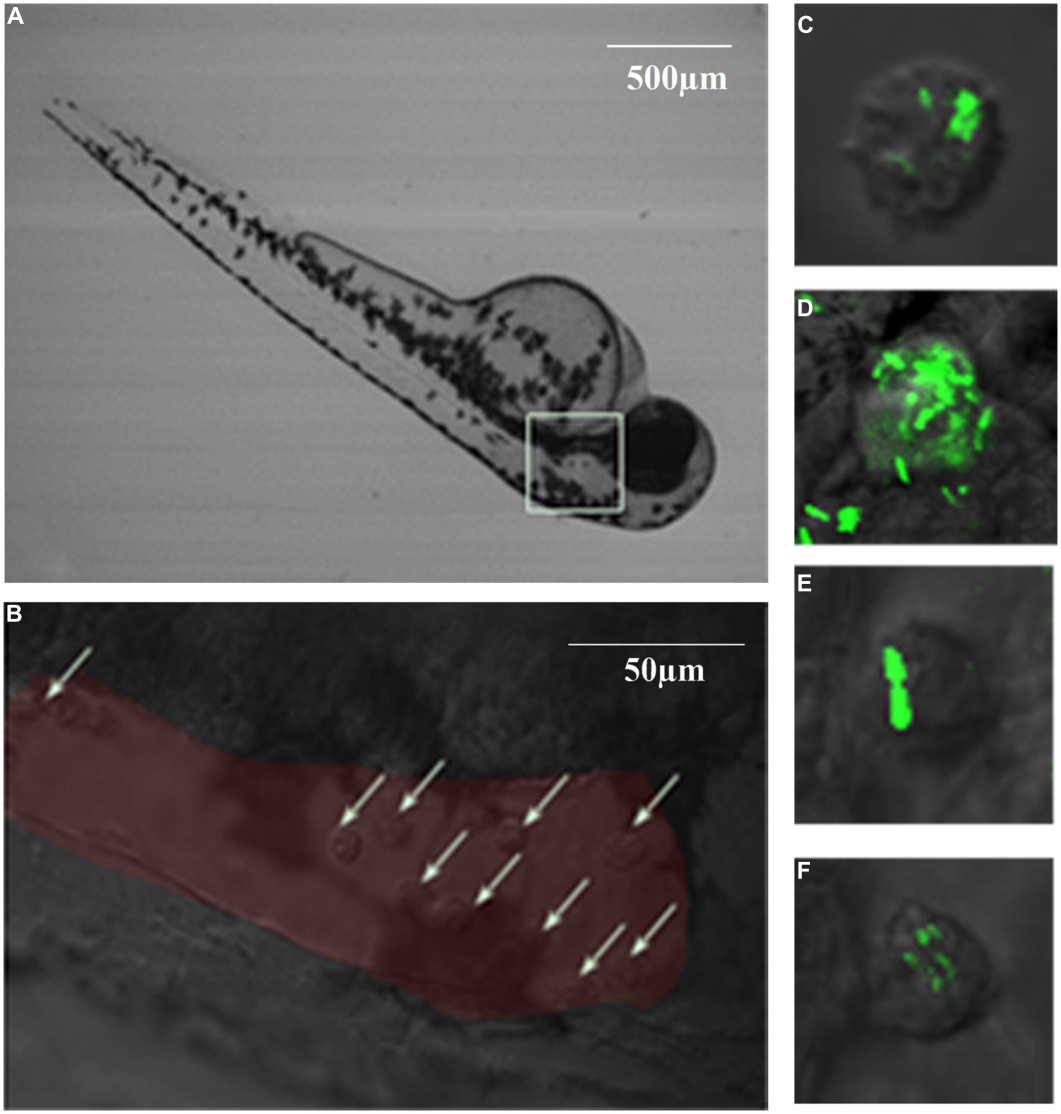
**Phagocytosis and bacterial proliferation in macrophages in 6 hpi germ-free zebrafish after brain ventricle injection with *L. monocytogenes* EGDe. (A)**: Injection site at low magnification; **(B)**: High magnification of the injection site (red area) showing recruitment of macrophages (white arrows) to the brain ventricle in response to bacterial infection; **(C–F)**: GFP-expressing bacteria were engulfed by macrophages.

Brain ventricle inoculation led to progressive lethal infection with two *L. monocytogenes* strains (**Figure 5**). However, EGDe showed more significant death than M7 at day 5 post-infection (dpi; 90 *vs.* 55%, *P* < 0.05). No further death was seen with M7, while there was no survival with EGDe at 10 dpi. Infection with *L. innocua* did not cause significant death, as compared with the mock infection. Macrophages at the infected site (**Figure 6A**, white box) were observed at 6hpi. Confocal microscopic examination showed that macrophages migrated to the brain ventricle (**Figure 6B**) and contained engulfed/attached bacteria (Figure 6C–F).

### Survival Rate was Dose-Dependent and Varied with Strains Upon Intravenous Infection

**FIGURE 7:**
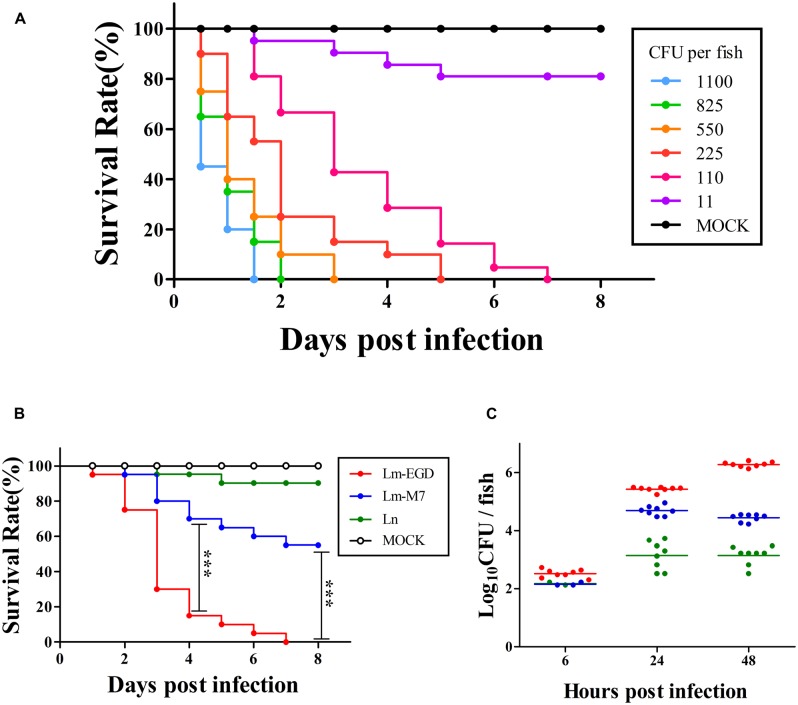
**Infection of germ-free zebrafish embryos, via intravenous injection, with graded inocula of *L. monocytogenes* EGDe (**A**, % survival with *n* = 20), or with similar inocula (80–140 CFU on average) of *L. monocytogenes* strains EGDe and M7 as well as *L. innocua* (**B**, % survival with *n* = 20; and **C**, bacterial proliferation with *n* = 8; and same color code for **B** and **C**).** ***P < 0.001.

**FIGURE 8:**
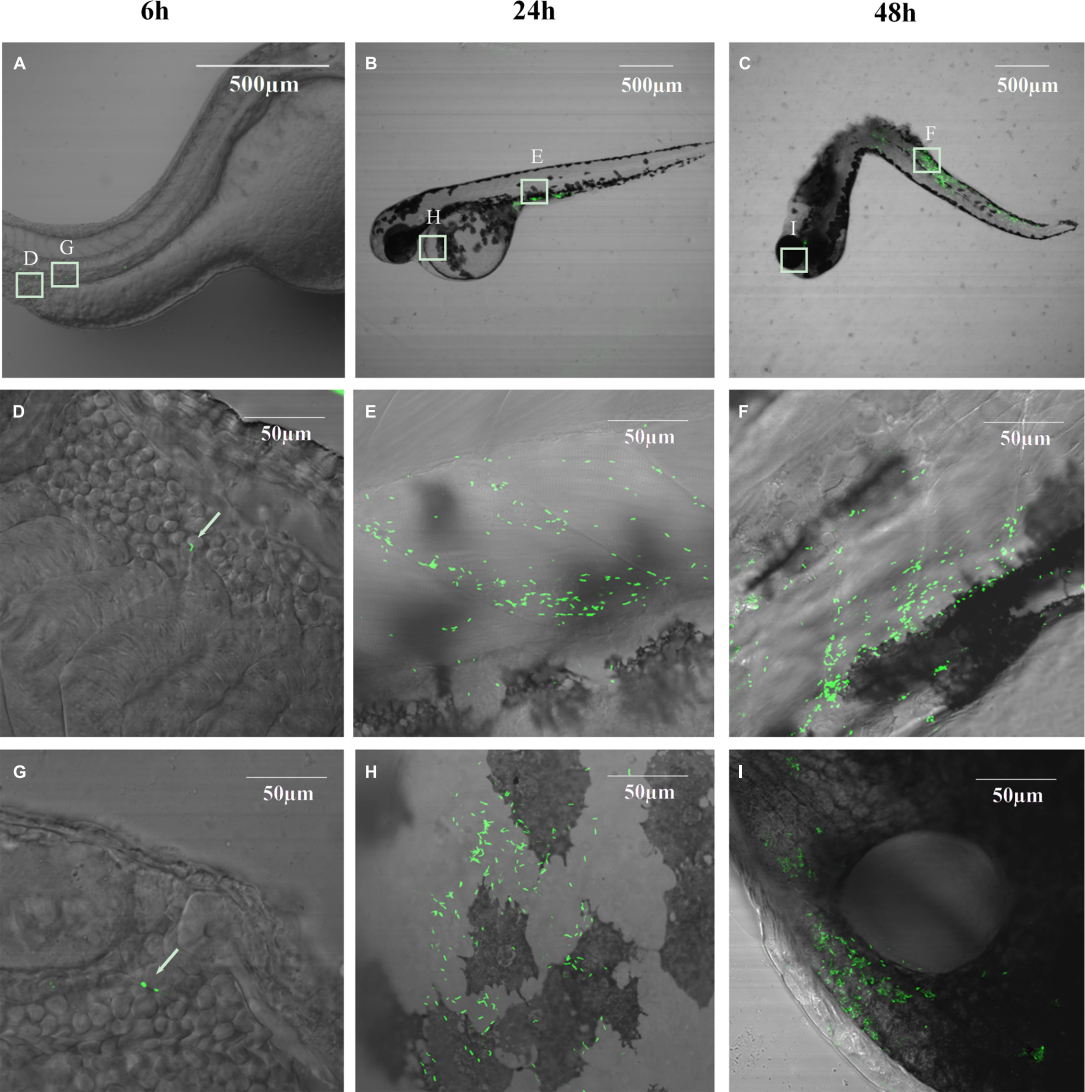
**Bacterial distribution and proliferation in germ-free zebrafish embryos infected with GFP-expressing *L. monocytogenes* EGDe carrying pFL251-gfp at 26 hpf via intravenous injection. (A–C)**: 6, 24, and 48 h post-infection at low magnification; **(D,G)**: Bacteria can be randomly observed at the injection site; **(E,F)**: Bacteria in trunk vessels; **(H)**: Bacteria in cardinal vessels; **(I)**: Bacteria in cerebral and retinal vessels.

**Figure 7A** shows that EGDe infection via the blood island resulted in progressive death with 100% mortality from day 2 to 7, depending on the inoculum level from 11 to 1100 CFU per fish. At similar inoculum levels (110 CFU per fish, ranges from 80 to 140 CFU), EGDe was far more pathogenic than M7 at 4 dpi (survival rate: 30 *vs.* 85%, *P* < 0.001) and at 8 dpi (0 *vs.* 55%, *P* < 0.001). *L. innocua* caused marginal death. By bacterial enumeration, we found that that there was initial growth of all inoculated strains from 6 to 24 hpi with EGDe having the highest bacterial load (**Figure 7C**). While the M7 strain and non-pathogenic *L. innocua* did not show further growth, the EGDe strain continued its growth at 48 hpi when fish death began (**Figures 7A–C**). To observe the bacterial load *in vivo*, we also infected the fish with GFP-expressing EGDe. **Figure 8** shows that EGDe strain had substantial proliferation in the fish over time. The bacteria could be randomly observed at the injection site at 6 hpi (**Figures 8D,G**), and then distributed in trunk and cardinal vessels at 24 hpi (**Figures 8E,H**). Substantial bacterial proliferation was seen in the trunk (**Figure 8F**) or in cerebral and retinal vessels (**Figure 8I**) at 48 hpi.

## Discussion

Stages of embryonic development of zebrafish have been well described since 1990s (Kimmel et al., 1995). The small transparent zebrafish embryos have been utilized as an alternative to mammalian animal models for studies of microbial infection and host responses (Cui et al., 2011; Meijer and Spaink, 2011; Novoa and Figueras, 2012). Previous work mostly focused on infections of conventional zebrafish embryos by individual routes of inoculation of their target microorganisms (Van Der Sar et al., 2003; Levraud et al., 2009; Volkman et al., 2010). A growing number of studies used the germ-free zebrafish to examine the immune responses to infections (Rendueles et al., 2012; Toh et al., 2013).

To ensure the sterility of germ-free zebrafish embryos we generated according to one of the methods described elsewhere (Pham et al., 2008), we monitored the total bacterial count in egg water and fish embryos on BHI agar up to 5 dpf. No bacterial colonies were seen. To further ensure that zebrafish embryos generated in our system were developing without the activation of innate immunity due to possible microbial colonization, we tested the expression of several Toll-like receptors in 5 dpf zebrafish embryos. Homogenized samples of germ-free and conventionally raised zebrafish were sacrificed for RNA extraction and RT-PCR. Five *tlr* genes (*tlr1*, *tlr2*, *tlr3*, *tlr4b,* and *tlr5b*) were selected from the list of 23 predicted zebrafish *tlr* genes (Meijer et al., 2004). Their transcriptional levels were barely detectable in zebrafish raised in the germ-free system, but highly induced in conventionally raised zebrafish (data not shown).

Initially we hypothesized that conventional zebrafish pro-colonized with commensal bacteria might render the fish resistant to immersion infection with pathogenic bacteria such as *L. monocytogenes*. We attempted to infect germ-free zebrafish larva to mimic the natural route of listeria infection through the digestive gut using the strains of different pathogenicity already known in other animal models (Schlech et al., 1993). Immersion inoculation to germ-free zebrafish at 5 dpf, a time with their mouth open and gut functional, did not cause any lethal infection even with the inoculum of the pathogenic *L. monocytegenes* EGDe as high as 10^10^ CFU/ml egg water. Lack of lethality was also seen in immersion infection with *L. monocytegenes* (Levraud et al., 2009) or *E. tarda* (van Soest et al., 2011) using conventional fish, while immersion infection of the conventional zebrafish with *E. ictaluri* led to high mortality (Rendueles et al., 2012). These results seem to indicate that the virulence determinants of the pathogens might be the key players in breaking through the gut barrier and cause systemic infection, and that deprivation of commensal microbes and lack of pre-stimulated immune responses do not predispose the fish to lethal infection.

To study if immersion could induce immune responses in infected fish larvae, several immune related genes were examined. *Cyp1a* is involved in intestinal epithelial detoxication and regulated by Toll-like receptor 2 in mice (Yamazaki et al., 2002; Do et al., 2012). *Irg1l* is homologous to mammalian *irg1* (immunoresponsive gene 1) which is expressed by macrophages and can be induced by proinflammatory cytokines (Degrandi et al., 2009). *Il1b*, a member of the interleukin 1 family of cytokines, is an important mediator of the inflammatory response (Barksby et al., 2007; Dinarello, 2009). *Mmp9* is a member of the matrix metalloproteinase (MMP) family that is important for remodeling of the extracellular matrix (Yoong et al., 2007). Oral immersion, though not lethal, induced expression of selected genes that peaked at 48 hpi but returned to normal at 72 hpi. It is clear that the pathogenic *L. monocytegenes* EGDe strain caused highest induction with nearly twice the transcriptional level of the low-pathogenic strain M7 and *L. innocua*. The latter two strains had similar induction levels in general, but significantly higher than the un-inoculated control. These findings suggest that stimulation of the innate immune responses was closely related to the virulence of the strains used.

Significant induction of *Irg1l*, *mmp9,* and *il1b* at 24 hpi was also seen from 25-hpf conventional fish immersion-infected with *E. tarda* (van Soest et al., 2011). However, we did not found such significant induction at this time point. Robust induction of IL-1β and TNF-α was observed as early as 2 hpi (TNF-α) or 4 hpi (IL-1β) in conventional zebrafish larvae immersion-infected with *E. tarda* at 24 hpf (Pressley et al., 2005). Besides different pathogenic species used, the microbial status may contribute to distinct responses between germ-free and conventional fish. Colonization of gnotobiotic 3 dpf fish larvae with conventional zebrafish microbeta induces NF-κB pathway activation in a dynamic temporal pattern with peak at 6 dpf (72 hpi; Kanther et al., 2011), about 24-h later than what we saw (48 hpi). Thus, it is tempting to speculate that priming of the fish with indigenous commensals at early age could facilitate the innate immune responses to invading pathogens. Bates et al. (2006) found that absence of microbiota during developmental stages of zebrafish was found to have arrested differentiation and altered function of macrophages.

Similar to our finding that expression of the innate immune genes returned to normal at 72 hpi, Pressley et al. (2005) observed even quicker decline of IL-1β at 12 hpi (from its peak at 4 hpi) in *E. tarda* infected fish by immersion. Such phenomena may indicate clearance of invaded pathogens by the macrophages. Herbomel et al have revealed that the embryonic macrophages of zebrafish are different from the adult, capable of proliferation and able to clear bacterial infection efficiently (Herbomel et al., 1999). Macrophages of zebrafish larvae could also control early *M. marinum* infection (Clay et al., 2007).

With injection inoculation, we found that the EGDe strain was far more pathogenic than the strain M7, as shown by significantly lower hatchability (yolk sac infection) and higher mortality (both intraventricular and intravenous infections). *L. innocua* strain did not show apparent lethality in all these infection routes. These results were consistent with murine models that shows the virulent nature of the EGDe strain (Brosch et al., 1993; Bécavin et al., 2014) and the low-pathogenicity of the strain M7 (Chen et al., 2010). This is in general agreement with major findings in a conventional zebrafish model showing that *L. monocytegenes* strain EGDe was more pathogenic than *L. innocua* (Levraud et al., 2009). However, the germ-free fish larvae were more susceptible to EGDe infection since the bacterial number causing 100% mortality within 2 dpi in our study was ∼10^3^ CFU, about 1 log less than that reported by Levraud et al. (2009).

The brain ventricle is a closed cavity which contains zero to two macrophages in zebrafish larva (Davis et al., 2002). Migration of macrophages into this cavity has been observed in several previous work examining the innate immune responses to infection (Herbomel et al., 1999; Clay et al., 2007). Besides the progressive lethality caused by brain ventricle injection with EGDe, we also observed macrophage migration to the brain ventricle and bacterial proliferation within the macrophages. This route of infection in the 26 hpf zebrafish embryos, a developmental period not hatched and without pigment formation, can serve as a good transparent model for visual observation of trafficking of phagocytic cells through the blood–brain barrier if the transgenic fish line (Li et al., 2012) is used.

In summary, our findings clearly indicate that immersion infection of the germ-free fish larvae induces distinct innate immune responses to *Listeria* strains of different pathogenicity. Since there is clear distinction of responses to infection between germ-free and conventional fish, possibly because of the confounding effects from the indigenous commensals in the conventional fish, the germ-free fish are preferred to examine immune responses to infection by pathogenic *Listeria* or other pathogens, such as trafficking of phagocytes and induction of inflammatory cytokines or genes related to innate immunity. Such work would help elucidate the molecular determinants of the host immune responses to infection and the virulence factors of the pathogen in initiating successful infection, when combined with readily available genetic manipulation technologies on both zebrafish and pathogens of interests.

## Author Contributions

WF, JP, and YS conceived and designed the experiments. YS, CF, CC, and YW performed the experiments. WF, JP, and YS analyzed the data. YS and WH wrote the paper.

## Conflict of Interest Statement

The authors declare that the research was conducted in the absence of any commercial or financial relationships that could be construed as a potential conflict of interest.
